# The Pretherapeutic Neutrophil-to-Lymphocyte Ratio for Docetaxel-Based Chemotherapy Is Useful for Predicting the Prognosis of Japanese Patients with Castration-Resistant Prostate Cancer

**DOI:** 10.1155/2019/2535270

**Published:** 2019-10-31

**Authors:** Tomoyuki Tatenuma, Takashi Kawahara, Narihiko Hayashi, Hisashi Hasumi, Kazuhide Makiyama, Noboru Nakaigawa, Takeshi Kishida, Yasuhide Miyoshi, Masahiro Yao, Hiroji Uemura

**Affiliations:** ^1^Department of Urology, Kanagawa Cancer Center, Yokohama, Japan; ^2^Departments of Urology and Renal Transplantation, Yokohama City University Medical Center, Yokohama, Japan; ^3^Department of Urology, Yokohama City University, Graduate School of Medicine, Yokohama, Japan

## Abstract

**Introduction and Objectives:**

The neutrophil-to-lymphocyte ratio (NLR) has been suggested as a simple marker of the systemic inflammatory response in critical care patients. The NLR can be easily calculated from routine complete blood counts in the peripheral blood. This parameter has been reported to be an independent prognosticator for some solid malignancies. In the present study, we examined the importance of the NLR as a prognostic marker for castration-resistant prostate cancer (CRPC) patients who received docetaxel- (DOC-) based chemotherapy.

**Methods:**

We analyzed a total of 73 patients who received DOC chemotherapy for CRPC in Yokohama City University Medical Center and affiliated hospitals. Complete blood cell counts were performed, and the NLR was calculated using the neutrophil and lymphocyte counts obtained on the same day or a few days before the initiation of DOC chemotherapy. We determined the NLR cutoff value based on the sensitivity and specificity levels derived from area under the receiver operator characteristic curves for death.

**Results:**

The median overall survival (OS) after DOC was 21.0 months (range: 2.0–51.0). The median OS was shorter in patients with a high NLR (≥2.59) than in those with a low NLR (<2.59) (12.0 versus 31.6 months, *p*=0.001). In the multivariate analysis, the NLR and lymph node (LN) metastasis were independent predictors of the OS (hazard ratio 3.643, *p*=0.001; hazard ratio 2.184, *p*=0.038, respectively).

**Conclusions:**

The higher NLR group showed a significantly poorer OS than the lower NLR group. Pre-DOC NLR might be a new marker for predicting the prognosis of patients who receive DOC chemotherapy.

## 1. Introduction

Prostate cancer (PCa) is the most common malignancy among men in the Western world [1]. In Japan, the morbidity of PCa has increased rapidly and will likely become the second-most common disease after lung cancer in 2020. We treat advanced and metastatic PCa with androgen deprivation therapy (ADT). However, while ADT is effective for advanced and metastatic PCa, the disease progresses to castration-resistant prostate cancer (CRPC) after a few years. The TAX-327 trial reported that docetaxel- (DOC-) based chemotherapy improved the overall survival (OS) of patients with CRPC [2]. DOC thus occupied an important position in the treatment of CRPC for a long period. However, enzalutamide and abiraterone acetate have become available for CPRC recently. In addition, the CHAARTED trial showed that early chemotherapy using DOC in addition to ADT in hormone-sensitive prostate cancer resulted in a significantly better OS than ADT alone [3]. The role of DOC-based chemotherapy is therefore changing and gaining increased significance.

The NLR has been suggested as a simple marker of the systemic inflammatory response in critical care patients. The NLR has been reported to be an independent prognosticator for some solid malignancies [4, 5]. This parameter can be easily calculated from routine complete blood counts (CBCs) in the peripheral blood. If the NLR can predict the efficacy of DOC-based chemotherapy, CRPC patients might be able to receive better sequential therapy. This study evaluated the utility of the NLR for predicting the prognosis of CRPC patients who received DOC-based chemotherapy.

## 2. Materials and Methods

### 2.1. Patients

A total of 73 CRPC patients were treated with DOC-based chemotherapy at Yokohama City University Hospital and Yokohama City University Medical Center (Table 1). All patients were castrated by surgery or medication, and their PSA subsequently increased consistently or the imaging findings showed disease progression.

### 2.2. Drug Administration and the Evaluation of Responses

DOC (55–75 mg/m^2^) was given every 3 to 4 weeks with 5 mg oral prednisolone or 0.5 mg dexamathasone twice daily. The dose of DOC was modified according to each individual patient's condition.

### 2.3. Clinical and Laboratory Assessment

Blood tests, including values for determining the NLR, were measured at the administration of DOC. The PSA response was defined as a decline of >50% in the PSA level from the pretreatment value. Disease progression was defined as an increase of >25% in the PSA level from the nadir and/or radiological progression according to the RECIST guidelines (version 1.1). The OS was defined as the time from the administration of DOC-based chemotherapy to death from any cause. Adverse events were graded by common terminology criteria for adverse events v4.0.

### 2.4. Statistical Analyses

The patients' characteristics and preoperative factors were analyzed by the Mann–Whitney *U* and one-factor analysis of variance (ANOVA) tests using the Graph Pad Prism software program (Graph Pad Software, La Jolla, CA, USA). Candidate cutoff points were detected using the area under the receiver operator curve (AUROC). The survival duration was defined as the time between the dates of the pathological diagnosis and tumor recurrence or death. A log-rank test was performed for comparisons between the higher and lower NLR groups. *p* values of <0.05 were considered to indicate statistical significance.

## 3. Results

### 3.1. Patients

The characteristics of the patients analyzed are shown in Table 1. A total of 73 CRPC patients were treated with DOC-based chemotherapy at Yokohama City University Hospital and Yokohama City University Medical Center. All patients were castrated by surgery or medication, and their PSA subsequently increased consistently or the imaging findings showed disease progression.

### 3.2. PSA Response

**Figure 1:**
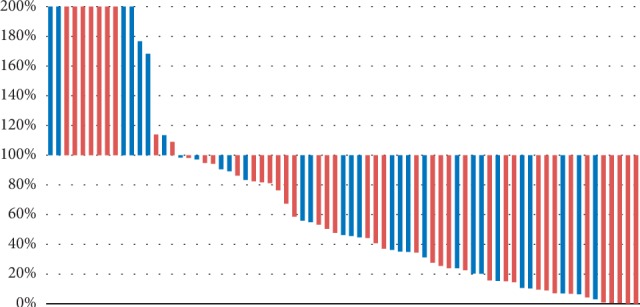
PSA differences and NLR value (red: NLR 2.59 or more; blue: NLR less than 2.59).

A PSA decline was observed in 78.0% of all patients, and *a* ≥ 50% decline in the PSA level from the baseline was observed in 53.4% of all patients (Figure 1).

### 3.3. Adverse Events

In the present study, 75.0% of all patients had at least one grade 3 or 4 adverse event. Neutropenia and febrile neutropenia was observed in 64.3% and 3.6% of all patients, respectively.

### 3.4. NLR Relevance to a Poor Prognosis

**Figure 2:**
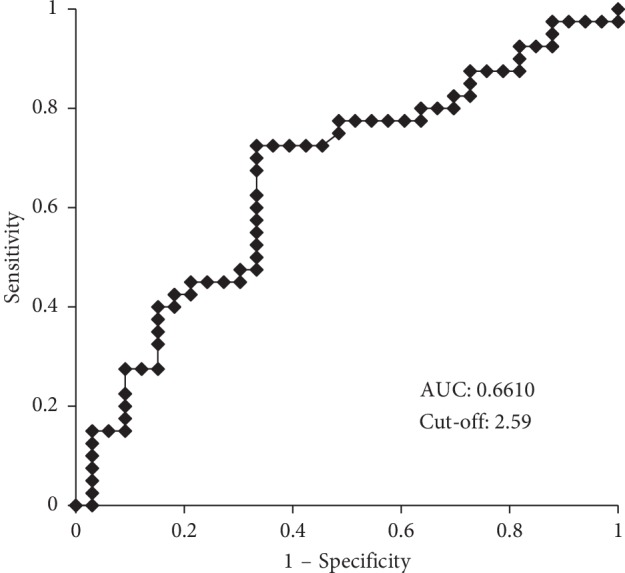
Candidate cutoff point was 2.59 and area under the curve was 0.6610.

**Figure 3:**
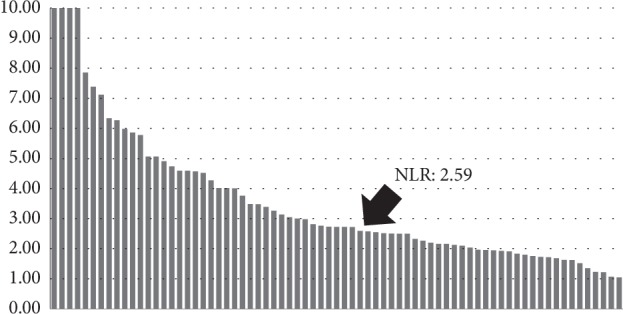
NLR value in patients who underwent docetaxel treatment.

**Figure 4:**
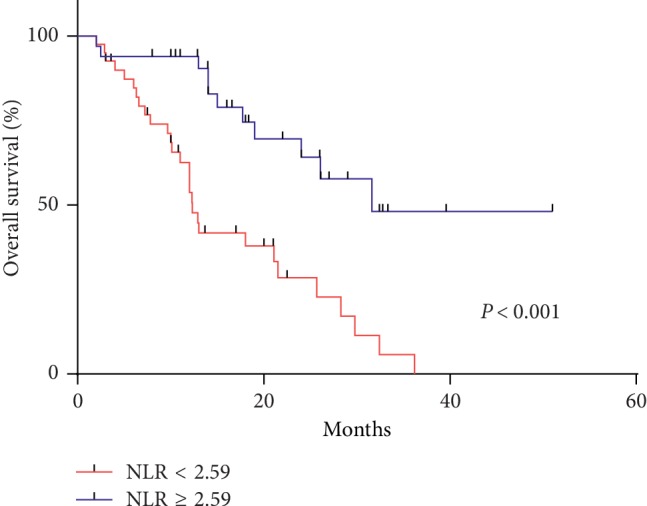
Kaplan–Meier analysis showed that higher NLR (2.59 or more) showed poorer overall survival (*p* < 0.001).

Based on the AUROC, the NLR cutoff point was determined to be 2.59 for death (AUROC: 0.6610; Figures 2 and 3). A Kaplan–Meier analysis and log-rank test revealed that a higher NLR was correlated with a significantly lower OS rate than a lower NLR (*p*=0.007; Figure 4).

### 3.5. OS after Starting DOC Therapy

For all patients, the median OS was 21.0 months. The lower NLR group showed a favorable OS compared to the higher NLR group (31.6 vs. 12.0 months, *p*=0.001; Figure 4). According to a multivariate analysis, lymph node metastasis and the NLR at the initiation of DOC were statistically significant predictive factors for the OS.

## 4. Discussion

With the recent development of new agents, including radium 223, cabazitaxel, enzalutamide, and abiraterone acetate, the landscape of CRPC treatment has been dramatically changing [6, 7]. However, DOC still plays an important role in both CRPC and metastatic hormone-sensitive prostate cancer (mHSPC) treatment. The FASTANA trial showed that DOC exerted the same efficacy as cabazitaxel. Furthermore, initial DOC treatment prolonged the OS in mHSPC patients, especially highly metastatic cases, as confirmed by the CHAARTED and STAMPEDE trials [3, 6].

Some previous studies have reported the prognostic factor in CRPC patients with DOC. Armstrong et al. and our previous study reported that organ metastasis, progression of bone metastasis, pain, and anemia were risk factors for a poor prognosis among patients who underwent DOC chemotherapy [8, 9]. Other studies have shown that the ECOG performance status (PS), serum lactic acid dehydrogenase (LDH) level, Gleason Sum, serum alkaline phosphatase (ALP) level, and serum PSA level are also potential candidate markers [10, 11]. Several prognostic systemic inflammatory markers, including the C-reactive protein level, albumin-based score, Glasgow prognostic score, and NLR, were reported to be correlated with the prognosis in CRPC [12, 13].

The NLR can be simply calculated from routine CBCs with differentials [14]. CBCs are typically performed during clinical checkups, and the NLR can be applied to virtually all patients either before or after surgery/medical treatment. It has also been reported to be an independent prognostic factor for several solid malignancies [4, 5, 15–23]. The NLR can be easily calculated from routine complete blood counts in the peripheral blood [20, 21].

Neutrophils play an important role in inducing cytokines for the progression and invasion of tumor cells. Elevated neutrophils result in tumor progression and induce metastasis [24]. Lymphocytes regulate the immune system, and reduced numbers of lymphocytes inhibit antitumor immunoreactivity and also result in a poor prognosis [25]. The NLR, which is a combination of the neutrophil and lymphocyte counts, reflects tumor progression and antitumor activity [26].

Some reports have shown that a higher NLR was associated with a poorer prognosis in CRPC patients undergoing DOC chemotherapy. Nuhn et al. reported that an NLR of <3 was associated with a significantly longer OS than an NLR of ≥3 (18.3 vs. 14.4 months, *p* < 0.001) [27]. Yao et al. also reported that an NLR < 3.5 was associated with a significantly longer OS and progression-free survival (20 vs. 15, *p*=0.013, 15 vs. 9.5 months, *p*=0.013, respectively) [28]. The OS of the present study was 25.6 months, while those of the TAX 327 and SWOG99-16 studies were 19.3 and 17.5 months, respectively. However, the patient backgrounds of the present and previous studies differed markedly, and our study had a relatively favorable outcome compared with the other studies.

There are several limitations associated with our study. First, the study was retrospective in nature. Second, the NLR may be affected by infection, systemic inflammation, and medication, although none of the patients in our study had such conditions at the time of blood tests before DOC chemotherapy. Third, the sample size was relatively small.

In conclusion, the higher NLR group showed a significantly poorer OS than the lower NLR group. The pre-docetaxel NLR might be a new marker for predicting the prognosis of patients who receive docetaxel chemotherapy.

## Figures and Tables

**Table 1:** Patients' characteristics.

		Number (%) or median (mean ± SD)			*p* value
		Total	NLR < 2.59	NLR ≥ 2.59	
Number of pts.		73	34	39	
Age		72 (70.3 ± 6.7)	72 (69.8 ± 6.4)	71 (70.8 ± 6.8)	0.444
Gleason score	6	2 (2.7%)	0 (0.0%)	2 (5.1%)	0.664
	7	7 (9.6%)	3 (8.8%)	4 (10.3%)	
	8	22 (30.1%)	12 (35.3%)	10 (25.6%)	
	9	27 (37.0%)	12 (35.3%)	15 (38.5%)	
	10	12 (17.8%)	6 (17.6%)	7 (17.9%)	
	Unknown	2 (2.7%)	1 (2.9%)	1 (2.6%)	
T stage	2	4 (5.5%)	4 (11.8%)	0 (0.0%)	0.096
	3	48 (65.8%)	21 (61.8%)	27 (69.2%)	
	4	12 (16.4%)	6 (17.6%)	6 (15.4%)	
	Unknown	9 (12.3%)	3 (8.8%)	6 (15.4%)	
Lymph node metastasis		57 (78.1%)	26 (76.5%)	31 (79.5%)	0.978
Bone metastasis		36 (49.3%)	15 (44.1%)	21 (53.8%)	0.552
Lung metastasis		8 (11.0%)	4 (11.8%)	4 (10.3%)	0.865
Liver metastasis		6 (8.2%)	3 (8.8%)	3 (7.7%)	0.801
Pre-DOC PSA		52.7 (315.1 ± 782.7)	30.1 (196.7 ± 529.9)	57.4 (412.8 ± 930.3)	0.196
PS	0	50 (68.5%)	23 (67.6%)	27 (69.2%)	0.734
	1	9 (12.3%)	3 (8.8%)	6 (15.4%)	
	Unknown	14 (19.2%)	8 (23.5%)	6 (15.4%)	
No. of DOC		8.0 (9.7 ± 7.8)	10 (10.6 ± 7.9)	7.0 (8.9 ± 7.7)	0.412
Hb		12.1 (11.8 ± 1.6)	12.3 (12.1 ± 1.3)	11.5 (11.4 ± 1.7)	0.036
ALP		726 (955.5 ± 903.2)	236 (596.0 ± 637.6)	841 (1135.2 ± 960.9)	0.160
LDH		217 (269.8 ± 167.4)	199 (250.4 ± 183.2)	233 (284.4 ± 148.6)	0.432

## Data Availability

Due to ethical restrictions, the raw data underlying this paper are available upon request to the corresponding author.
